# Lectures on Clinical Medicine, Delivered at the Philadelphia Medical Institute

**Published:** 1838-05-09

**Authors:** W. W. Gerhard

**Affiliations:** Physician to the Philadelphia Hospital, &c.


**LECTURES ON CLINICAL MEDICINE,** *delivered at the Philadelphia Medical Institute, by* W. W. Gerhard, M. D., *Physician to the Philadelphia Hospital,* &c.

RUBEOLA.

*Tuesday, April 24th.* Dr. Gerhard commenced the lecture, by calling attention to the case of the man Robb, which was under notice, on the subject of *acute articular rheumatism.* The opiate practice had been carried out, with decided but gradual improvement. Yesterday, however, there was a return of the affection, but under a much less severe type, marking the stage following the acute form of the disease, in which the symptoms are swelling and mere soreness, rather than pain or heat. With the reappearance of the affection in this modified shape, the impulse of the heart, which had been increasing, has been, for two days, much diminished. There is at present an effusion of about a pint of liquid into the pericardium. The dullness on percussion is so manifest, as to leave no doubt of this fact. It is not from the simple presence of unusual dullness, that we draw our conclusion, but because we have seen this dullness notably increase, from day to day, since the patient has been under inspection. A prominence of the chest over the region of the heart has also appeared, in a marked manner, during this time. Another proof of the effusion is the absence of the impulse of the heart, which is next to nothing. You recollect the roughness of the two sounds of the heart, particularly the first; this roughness has diminished, as effusion has gone on. In proportion then, as the pericarditis has advanced towards secretion, have the signs of endocarditis become less evident. This fact, which exemplifies a general rule of pathology, is worth recollecting. Of pericarditis the physical signs are, mainly, the increase of prominence and dullness, with faintness of the impulse of the heart, while endocarditis is to be recognised by increased action of the organ, and the roughened sound, sometimes amounting to that, termed rasping. The physical signs of these two affections, particularly of pericarditis, are exceedingly easy of recognition, so much so, that, knowing what they are, you will hardly fail to detect them. The two diseases are not likely, I have told you, to exist together, under an equally severe type. The same thing is true with pleurisy and pneumonia; they may co-exist, but very severe pleurisy and pneumonia do not go together. If, for example, the pleurisy be aggravated, by the compression of the lung, it prevents the development of acute inflammation. The law of pathology, founded on the two cases I have adduced, you will find generally to hold good. I shall here conclude my remarks on the case of rheumatism: the opiate practice was continued till yesterday, when it was modified by the substitution of a single dose of Dover's powder at night, in place of the opium pills.

It is my intention in this course of lectures, gentlemen, to take up the various acute diseases in succession, as they come before our notice, at the hospital, preserving, as far as possible, the natural connection amongst them. A very unusual affection in general hospital practice has lately claimed your attention, I mean *rubeola.* To see it pervading epidemically the wards of adults is a phenomenon, which I have never before witnessed, and hardly expect again to observe; as it is a disease which usually appears but once during life, and is generally confined to childhood. During the last six weeks, however, there have been as many as seven or eight cases, in my single service, and three or four in the other wards. My recent cases were as follows:

Morris, a man of nearly forty; Perry, a lad of eighteen, and three others nearly of the same age.

Previous to detailing the symptoms which characterize rubeola, I shall make a few remarks on its pathology. The pathology of measles, like that of other exanthematous affections, is to be divided into two parts, one comprising the morbid changes in the body, which are characteristic of and essential to the disease, the other being those which are merely accidental. The first are of course to be looked on as pathognomonic.

The description of the affection, given by Sydenham, is so good, and agrees so accurately with its appearance, at the present day, that I shall read it to you at length, and adopt it, in most particulars, in preference to more modern accounts. It cannot be amended materially, except by examining the symptoms with the aid of the numerical method; a task which, at present, I am not able to undertake.

This excellent description of Sydenham's shows his powers of observation in favourable contrast with some of modern times. His general account of the disease holds good in the cases, which we observed at the hospital. Thus, our symptoms of the first day, like his, were chilliness and cold shiverings. The second day we had the catarrhal symptoms, connected with coryza and the flow of tears, as described by Sydenham. This is the best sign to distinguish measles, in its incipient stage, from other exanthemata. In this stage, the other exanthematous affections offer no mark by which they can be diagnosticated with any certainty. They have, at this time, numerous symptoms in common, including some belonging to other febrile diseases. Thus, in scarlatina, the sore throat is by no means sufficiently characteristic, and small pox may, at its commencement, be very readily mistaken for typhoid fever. Dr. Louis, who certainly is most accurately familiar with typhoid fever, has more than once mistaken for it the incubation of small-pox.

The symptoms that follow, as the sick stomach, loss of appetite, slight cough, heaviness of the head and eyes, occur now just as they did in the time of Sydenham. The only irregularity in Sydenham's description consists in the large red wheals, which have not, in our cases, made their appearance, nor have I often observed them. The swelling of the eye-lids continues the same. The vomiting occurs particularly in children, and not in adults; we did not notice it in these cases.

Looseness of the bowels is the next symptom mentioned. This is not now a constant symptom in the early stages of measles, but, it is to be recollected, that the descriptive account of the disease by Sydenham has reference to an epidemic which took place in 1670. The diarrhœa I set down as an accidental symptom, and, as such, it probably complicated the epidemic of that year.

We next pass to his description of the eruption, which he characterizes most accurately. We have it now as then, appearing first in the form of red spots, resembling fleabites, which gradually coalesce into semicircular, crescentic, and circular shapes, showing themselves first on the face, and spreading thence over the rest of the body. As the eruption increases, there is a diminution of the other symptoms. The eruption is found in the mouth and throat, as well as on the skin. In the cases of the negroes, it was of course detected only in the eyes and throat. In the pharynx and palate, as elsewhere, the eruption was not so much elevated above the epithelium, as it is above the surface of the skin. The next part of the description is doubtful—that is, the mode of disappearance of the eruption. It does not disappear on the eighth or ninth day, as alleged by Sydenham, for traces of it remain for some time afterwards, in copper-coloured spots, as shown in the cases in our wards; even after the spots entirely disappear the skin remains rough and dry. I do not at this time intend to go more largely into the ordinary symptoms of measles, for I can scarcely add any thing to the graphic description which I have read to you from Sydenham. While at Paris, and at the Hospital des Enfans Malades, I collected a mass of observations on this subject; but, not yet having been able to analyse them, I must defer presenting them to you to some future time. I shall now call your attention to two of the accidental symptoms which may complicate the regular course of measles and often become the sources of great danger.

The first is bronchitis, of a severe character. A slight bronchitis may be looked upon as a necessary symptom of the disease; it is to be deemed accidental, when it appears under an aggravated type, or when the inflammation runs into the parenchyma of the lungs, and takes on the form of lobular pneumonia, which is similar to the pneumonia following the bronchitis of young children. This accidental symptom occurred in the man Morris, whom you recollect in the first ward; about the eighth or ninth day, when the eruption was fading, and our attention was directed to the development of moist rhonchi on the right side of the chest, showing the existence of severe bronchitis, with considerable dulness on the middle and posterior part, and some on the anterior region, of the left side—a common seat of lobular pneumonia in measles. Instead of getting well, the man has remained ill, in this state, exemplifying the general rule, that, when lobular pneumonia is developed, after the subsidence of the eruption, it lasts for a considerable length of time. The signs, by which its appearance is to be detected, are dulness on percussion, with a sub-crepitant rhonchus and a slightly bronchial respiration. In place of attacking the mass of the lung, and rendering it solid, the inflammation appears in the isolated lobules, leaving amongst them portions of the lung still permeable to the air, which prevent the development of loud bronchial respiration. The respiration, in the very early stages of the disorder, and in the portion of the lungs, which are not inflamed, is not lost, but rendered louder, and roughened.

In the case of the boy Perry, the pneumonia appeared on the eleventh day of the disease, after the eruption had entirely subsided, no traces of it being left but a few copper-coloured spots. His right lung was attacked, as is commonly the case; perhaps, from its greater size, and from the circumstances of the patient's lying upon the right side. The lower, and not the middle and upper lobes, was attacked; in this respect as well as in others, it is like ordinary pneumonia, but differs from it, in the loudness and looseness of the crepitus, which ceases in regular inflammatory pneumonia, as soon as the entire substance of the lungs becomes solidified. In this boy's case, as in that of Morris, convalescence has been very slowly established, and is yet by no means perfect; he is still lingering in a somewhat critical condition. In the case of Morris, I entertained, for a time, some fear of the existence of tubercles, the development of which is thought to follow attacks of measles; I say, is thought, for I am by no means certain that there is any necessary connection between the two affections.

The treatment proper to meet this complication of measles, is necessarily various. At the hospital des Enfans Malades, during my residence, local depletion by cups and leeches was largely employed by Dr. Guersent. But the debility, consequent on this mode of treatment, was favourable to the reproduction of the disease in other parts of the lungs, especially as the pneumonia was observed almost invariably in children of feeble constitution. The proper rule for the employment of leeches, is to confine it to cases, in which there is excessive dyspnœa, and a rapid extension of the pneumonia is going on. It extends through the lung most rapidly, in stout, robust children, and in them leeching does good. In the ordinary lobular pneumonia, as well as in that which follows measles, after one or two cuppings, the best treatment consists in small doses of ipecacuanha. By persevering with this remedy, until the expectoration, or rather the secretion, (for with children there is no expectoration, as they swallow the discharge,) is freer, the patient is relieved, and we may then complete the cure, by the exhibition of tonics and a generous diet. Above all, attention is to be directed to position. If the child lie constantly on its back, the development of pneumonia is almost certain. It must, therefore, be moved frequently from one side to the other, and be from time to time raised in bed or carried about. In addition to ipecacuanha in expectorant doses, the sulphate of quinine and some preparation of iron, in small quantities, may be given, combined with a generous diet, if the child should become feeble, and the quantity of red blood should diminish. You will find, that in my lectures, gentlemen, I am not at all disposed to insist on too rigid a diet. I have seen so much mischief result from the continued enforcement of a rigid diet, in the mode of practice which was prevalent in France a few years ago, that it is with great caution, and no little fear, that I venture upon it, except for a short period. In some of the wards of the Enfans Malades, the practice was to place the children on a rigid diet, and the results were certainly far from favorable.

In the cases under notice, by pursuing the practice indicated, we have in a great measure, succeeded in getting rid of the accidental symptoms. But there is still some cough, and other traces of lingering bronchitis. What is now the proper treatment? It should be principally hygienic. The patients are to go freely into the open air, taking internally, at the same time, some of the milder tonics.

The next accidental symptom, likely to complicate the course of measles, is severe diarrhœa, near to the close or after the termination of the disease. At the Enfans Malades, the children died in two ways, when measles proved fatal, of lobular pneumonia, during the active period of the affection, and of diarrhœa, at the end of it. The lobular pneumonia usually showed itself at about the sixth day, the bronchitis appearing much earlier; but the diarrhœa did not come on, until the eruption was almost over, and desquamation was taking place. If this diarrhœa be but slight, no danger need be apprehended from it, and we rather avoid much interference with it. Indeed, it is generally looked upon as a safeguard to the child, and is, therefore, suffered to run on. But I do not consider the diarrhœa as slight and not to be checked, if it exceeds four, five, or six stools, during the day, and continues until it is accompanied by emaciation of the child, with paleness and dryness of skin. This variety of diarrhœa depends upon a particular state of the mucous membranes, in which they are pale and soft, seeming to be acted on by the altered fluids in the body, and instead of being themselves the seat of very active disease. I showed you the other day, at an autopsy, a similar state of the mucous membrane, but occurring in the stomach; in this case, however, it was probably produced by the action of the fluids, after death. This state of the mucous membranes, as it occurs in measles, I do not regard as an effect of inflammation, nor is it to be treated as such. Depletion, of any sort, here does no good, nor do remedies specially directed to the bowels always prove of much service. You must act on the skin, until its functions are restored, and for this purpose nothing is better than the sulphur bath, made by dissolving the sulphuret of potassa in water. I have seen children recover, at the Enfans Malades, under this treatment with astonishing rapidity. It not only relieves the particular symptom, to which it is addressed, but much improves the general condition of the patient. Indeed, it was remarked by Jadelot, that, the same remedy, employed for the management of itch, not only cured that affection, but besides left the patient in a general state of health and embonpoint. If the sulphur bath cannot be administered, one of warm salt water may be substituted. In addition to this treatment, adapted to the skin, slight opiates may be resorted to, with small doses of ipecacuanha, and astringents, which are supposed by some to act chemically upon the bowels. But depletion, by leeches or cups, must be abstained from, and the diet must be nutritious.

The last variety of accidental lesion, which occurs during measles, is acute diarrhœa during the height of the affection. This complication we have not witnessed during the epidemic at the hospital, though it was a very frequent occurrence, at the Enfans Malades, in 1832, which was just before the cessation of the Asiatic cholera at Paris. This epidemic of measles was probably similar in its character to that described by Sydenham. It is dependent on acute inflammation of the colon, and shows itself at the most severe period of the eruption; it is attended, generally, with the usual symptoms of dysentery, considerable pain, stools of small quantity containing slime, sometimes patches of false membrane, and blood; in fact, we have a regular attack of acute dysentery, complicating the measles. This complication is, I believe, most apt to occur in the summer months of the year. That is, measles are subject to the general rule of pathology, which determines the nature of the accidental symptoms, attending self-limited diseases. Thus, in the typhus fever, which was epidemic here during 1836, and part of 1837, we had, during the winter, symptoms of the acute affections most usual in winter, as those of the chest, and, in summer, it was complicated with diseases which are endemic in hot weather, as dysentery and disorders of the alimentary canal. Neither of these affections was in any manner a necessary accompaniment to the typhus. The complication of measles follow the same rule, except that, both the inflammations of the lungs and the bowels are more frequent than in typhus fever; we have, in other words, very generally lobular pneumonia occurring in the measles of winter and early spring, and affections of the alimentary canal, when the epidemic takes place in the summer months, particularly July and August.

The post mortem appearances, in this affection, differ from those of ordinary diarrhœa. If closely examined, the colon and rectum are found to be covered with patches of lymph, and their mucous membrane is much disorganized, and of a violet tint, as in severe dysentery. So universal were these appearances on dissection, during the epidemic at the Enfans Malades, to which I have just alluded, that a gentleman, who was observing it, thought that he had discovered a new law of pathology, and that there was a constant connection between rubeola and inflammation of the colon. He was, however, mistaken, and from his mistake we may infer the importance of observing with care the phenomena of several epidemics, and of again and again repeating these observations, before we allow ourselves to make from them any general deductions.

The treatment, at the Enfans Malades, for this dysenteric affection was the same that is employed in ordinary dysentery. It was attended with no great success, but it must be remembered, that severe dysentery is at all times a difficult affection to treat. The remedies, however, should certainly be the same in the complication we have been speaking of, as in the common variety. In the early stage, we must have recourse to antiphlogistics, with some fearlessness, by leeches and cups to the region of the colon and the anus. This dysentery differs essentially, as I have before said, from the diarrhœa, occurring at the close of measles, and we are to have no fears here about the propriety of an energetic antiphlogistic treatment; it affords prompt and great relief. We may afterwards administer opiates in very small quantities; and moderate doses of ipecacuanha. Calomel is so rarely employed in France, that I have never seen it prescribed in these cases, and have not been able to test its efficacy in this affection, frequently enough to speak of the advantages of using it. The after management of the dysentery of measles is much the same as in common dysentery, except that the former will be found to be of greater obstinacy than the latter usually is.

From these details, then, we deduce the following corollary. In measles, as in other diseases of known duration, we have one constant set of symptoms, as the eruption, and febrile movement with anorexia, thirst, restlessness, &c.; and next, a series of accidental symptoms, which extend from the slight bronchitis, necessary to the affection, to severe bronchitis and lobular pneumonia, and from the slight necesary diarrhœa to diarrhœa of the sub-acute form, and severe inflammatory dysentery. It is to these accidental symptoms that you are to pay particular attention; and by doing so, I am persuaded you will much diminish the mortality of measles, which depends, as in typhus fever and small-pox, on the severity of the accidental complications.

There remain to be noticed some varieties of measles, not observed here in the late epidemic. The first variety may occur in the other exanthemata, and consists in an imperfect development of the eruption. This is not so frequent in measles as in scarlatina; but we have occasionally coryza, a flow of water from the eyes, and cough, with but a very slight eruption, or one that is confined to the face. This is still a genuine, although an anomalous form of measles.

The second variety consists in the severe complication of internal inflammation with an eruption, which disappears soon after the beginning of the disease, and may be looked upon as suppressed. You will have universal bronchitis, the whole mucous membrane being affected with inflammation of an intense character, instead of the usual slight blush. We have then a grave internal affection, occasioned by the want of action on the surface of the body, the disease being, as it were, concentrated in the internal organs. This variety is always attended with great danger. It is to be treated by active counter-irritation of the skin, to supply the place of the absent eruption; for this purpose, sinapisms, the warm bath, and the like remedies are to be resorted to.

The third variety is the black measles, or rubeola nigra. This is no real variety. It occurs in feeble children, in whom the blood is in a dissolved state, as from scurvy; or it may depend on the sudden development of lobular pneumonia, preventing the proper decarbonization of the blood in the lungs, and giving it a general dark tint.

These varieties are almost the only ones that you will meet with in practice, and on which it is therefore proper to dwell. Rubeola sine catarrho I have never seen, nor do I believe in its existence. Some change in the bronchial mucous membrane is always to be detected; there is a dry rhonchus indicating a thickening of it, or we have at least some traces of a moist secretion. Cough is not a necessary attendant upon a slight bronchitis. It is impossible to decide with certainty upon its non-existence without a very careful examination, and I suspect it is the absence of close observation that has given rise to the variety of rubeola sine catarrho.

I have presented to you to-day but few clinical illustrations, as I was desirous of giving you a somewhat detailed descriptive notice of measles, a disease of so frequent occurrence, and which now prevails epidemically. I have insisted particularly upon the importance of the accidental symptoms which are most frequent, although other organs, as the brain and the wind-pipe are sometimes the seat of grave lesions, but they are not usually so much affected as the thoracic and abdominal viscera. There is another complication which is not rare in some epidemics, that is the gangrenous sore mouth of children, of which I shall treat at a future time.

Measles is perhaps a more frequent cause of after ill health than any of the other exanthemata. The bad effects of small-pox and scarlatina are usually confined to the course of the disease; they destroy life at this time or soon after. But measles, though less dangerous, during the eruption, may leave behind it greater organic lesions than either of the others. The effects of lobular pneumonia and diarrhœa are not easily got rid of; and, after a supposed convalescence from measles, we but too often see our little patients wasting away from emaciation, and, after a lapse of a few months, perish from the consequence of one or other of these dangerous complications.

